# Ruptured sinus of Valsalva aneurysm: Yet another hole to plug!

**DOI:** 10.4103/0974-2069.52818

**Published:** 2009

**Authors:** Prafulla G Kerkar

**Affiliations:** Department of Cardiology, King Edward VII Memorial Hospital, Parel, Mumbai - 400 012, India

Ruptured sinus of Valsalva aneurysm (RSOV), a rare but well- recognized clinical entity, is invariably a form of left-to-right shunt due to rupture into right-sided chambers. It causes profound hemodynamic effects, especially when the rupture is acute. Like most other left-to-right shunts, it was only a matter of time before this rare defect also became amenable to transcatheter closure (TCC). Since the first report of TCC of RSOV by Cullen *et al*. in 1994[1] using the Rashkind umbrella, in recent times, there have been a spate of case reports, brief communications and interesting case presentations at interventional meetings using the much user-friendly and effective Amplatzer devices. Given the preponderance of RSOV in Asians compared with the Western population,[2] it was only natural that the first series of eight patients was reported from India by Arora *et al*.[3] In this issue of the journal, Sen *et al*.[4] from India report another series of eight patients performed in a short span of 4 years a commendable effort. Encouraged by the excellent 1-year outcome of TCC of RSOV in our first patient, a post-CABG, critically-ill patient,[5] we too have attempted TCC of RSVA in 17 patients between July 2004 and Jan 2009.[6] Traditionally, surgical closure has been the mainstay of therapy for RSOV, with an operative mortality of <5 %.[2] Before offering TCC of RSOV as an acceptable alternative to surgery, we need to ponder over (i) the criteria for case and device selection, (ii) the nuances of the technique and (iii) the possible complications.

## CASE AND DEVICE SELECTION

Sen and coworkers[4] attempted TCC in patients with isolated RSOV only. However, TCC of RSOV and atrial septal defect (ASD) in the same patient has been reported recently.[7] We have offered TCC of RSOV to patients with associated defects that were also amenable to transcatheter therapy. One of our patients underwent coarctation stenting at the same sitting whereas another patient had TCC of secundum ASD at a second session. On the other hand, we excluded patients with associated ventricular septal defect (VSD), although it is conceptually possible to perform TCC of VSD as well in the same patient. Other exclusion criteria would be associated aortic regurgitation (AR) requiring surgery, large RSOVs and RSOV with multiple sites of rupture. However, Feldson *et al*. have performed TCC for RSOV rupturing into the right atrium and the right ventricle using two Amplatzer duct occluders (ADOs).[8] Because RSOVs are commonly “wind-sock”-like, with a broader aortic end, ADO is best suited for this defect, although other Amplatzer devices[9,10] as well as coils[11] have been used by others. It is important to rule out infective endocarditis as a possible etiology for the acute presentation of the patient with RSOV.

## TECHNICAL DETAILS

The technique is very similar to TCC of perimembranous VSD, given that the defect is just above the aortic valve instead of below. It entails crossing the defect from the left side, establishing the standard arteriovenous wire loop and deploying the device from the venous side under fluoroscopic and transesophageal echocardiographic (TEE) guidance. Although Arora *et al*.[3] have used transthoracic echocardiography, we believe TEE is invaluable for its excellent delineation of the anatomy, both pre-procedurally and during the device deployment, and to monitor the occurrence of AR. Moreover, it helps in limiting angiography and its attendant contrast load in critically-ill patients. It is believed that three-dimensional TEE may provide a more accurate measurement of the defect size and position.[12] Balloon sizing of the defect is not necessary, although used by some.[3] We have used an ADO 2-4 mm larger than the aortic end of the RSOV, as measured on TEE/angiography. Upsizing the device may be necessary at times because the margins of the defect are quite flimsy (“wind-sock-like”). Attempts should be made to close the defect as far as possible near the aortic end similar to a “surgeon's repair” to avoid leaving behind any uncovered residual aneurysm with a potential to rupture at another site. In such attempts, it is essential to look for any encroachment of the device on the aortic valve causing significant AR on TEE.

## COMPLICATIONS

Residual shunting causing hemolysis in this high-flow defect and procedure-related AR are the potential complications of TCC of RSOV. Although Sen *et al*.[4] have had no complications, residual shunting and severe hemolysis requiring repeat intervention and eventual surgery has been described.[3] We have also encountered significant hemolysis in one patient with a large RSOV closed with a 16/14 mm ADO, which abated on conservative treatment. At 1-year follow-up, there was no residual shunt in this patient. Procedure-related significant AR before device release necessitated retrieval and surgical referral in one of our patients. In four other patients, there was trace AR, which disappeared on follow-up in two. It is imperative to critically assess AR before, during and after the procedure to draw meaningful conclusions about TCC of RSOV because the presence of post-operative AR at discharge is the single most important determinant of long-term outcomes following surgery for RSOV.[13] Although encroachment of the device on the coronary arteries is carefully looked for, it has never been reported and is a non-issue. However, due to the proximity of the coronaries, we empirically use dual antiplatelet therapy periprocedure.

In summary, RSOV is yet another hole that can be successfully plugged by the interventional cardiologist. TCC can be considered a safe and effective alternative to traditional surgical correction in selected cases. However, a longer follow-up is necessary. Given the rarity of this defect, it would be essential to maintain a registry of such procedures.

## References

[1] Cullen S, Somerville J, Redington A (1994). Transcatheter closure of ruptured aneurysm of sinus of Valsalva. Br Heart J.

[2] Chu SH, Hung CR, How SS, Chang H, Wang SS, Tsai CH (1990). Ruptured aneurysms of the sinus of Valsalva in Oriental patients. J Thorac Cardiovasc Surg.

[3] Arora R, Trehan V, Rangasetty UM, Mukhopadhyay S, Thakur AK, Kalra GS (2004). Transcatheter closure of ruptured sinus of Valsalva aneurysm. J Interv Cardiol.

[4] Sen S, Chattopadhyay A, Ray M, Bandopadhyay B (2009). Transcatheter device closure of ruptured sinus of Valsalva: Immediate results and short term follow up. Ann Pediatr Card.

[5] Kerkar P, Suvarna T, Burkule N, Panda R (2007). Transcatheter closure of ruptured sinus of Valsalva aneurysm using the Amplatzer duct occluder in a critically ill post-CABG patient. J Invasive Cardiol.

[6] Kerkar P (2009). To be presented at 5th World Congress of Pediatric Cardiology.

[7] Cui W, van Bergen AH, Patel D, Javois AJ, Roberson DA (2008). Transcatheter closure of ruptured sinus of valsalva aneurysm and secundum atrial septal defect with limited inferior rim. Echocardiography.

[8] Fedson S, Jolly N, Lang RM, Hijazi ZM (2003). Percutaneous closure of a ruptured sinus of Valsalva aneurysm using the Amplatzer duct occluder. Catheter Cardiovasc Interv.

[9] Cullen S, Vogel M, Deanfield JE, Redington AN (2002). Rupture of aneurysm of the right sinus of Valsalva into the right ventricular outflow tract: Treatment with Amplatzer atrial septal occluder. Circulation.

[10] Schaeffler R, Sarikouch S, Peuster M (2007). Transcatheter closure of a ruptured sinus of Valsalva aneurysm (RSVA) after aortic valve replacement using the Amplatzer muscular VSD Occluder. Clin Res Cardiol.

[11] Rao PS, Bromberg BI, Jureidini SB, Fiore AC (2003). Transcatheter occlusion of ruptured sinus of Valsalva aneurysm: Innovative use of available technology. Catheter Cardiovasc Interv.

[12] Jean WH, Kang TJ, Liu CM, Chang CW, Tsai SK, Wang JK (2004). Transcatheter occlusion of ruptured sinus of Valsalva aneurysm guided by three-dimensional transesophageal echocardiography. J Formos Med Assoc.

[13] Murashita T, Kubota T, Kamikubo Y, Shiiya N, Yasuda K (2002). Long-term results of aortic valve regurgitation after repair of ruptured sinus of valsalva aneurysm. Ann Thorac Surg.

